# Parathyroid Hormone is Negatively Correlated with Glycated Hemoglobin in Newly Diagnosed Type 2 Diabetic Patients

**DOI:** 10.1155/2024/8414689

**Published:** 2024-04-01

**Authors:** Xueqing He, Man Liu, Xiaoyu Ding, Nannan Bian, Jiaxuan Wang, Guang Wang, Jia Liu

**Affiliations:** Department of Endocrinology, Beijing Chao-Yang Hospital, Capital Medical University, Beijing 100020, China

## Abstract

**Objective:**

The growing evidence shows that parathyroid hormone (PTH) may affect glucose metabolism. However, the relationship between them is still controversial among diabetic patients. The current study aimed to investigate the relationship between PTH and glucose metabolism in the patients with newly diagnosed type 2 diabetes (T2D).

**Methods:**

A total of 532 participants, including 387 patients with newly diagnosed T2D and 145 healthy controls, were recruited in the present study. PTH and metabolic parameters were measured in all participants.

**Results:**

The PTH levels were significantly lower in the newly diagnosed T2D patients compared with the control group (35.10 (25.90, 47.20) vs. 47.15 (35.83, 58.65) pg/ml, *P* < 0.001). The T2D patients with a higher glycated hemoglobin (HbA1c) tertile had lower PTH levels than the patients with a lower HbA1c tertile (32.90 (24.85, 41.40) vs. 37.50 (26.10, 54.55) pg/ml, *P* < 0.001). Spearman correlation analysis showed that PTH was positively correlated with the body mass index (BMI), fasting insulin (FINS), homeostasis model assessment of *β*-cell function (HOMA-*β*), and homeostasis model assessment of insulin resistance (HOMA-IR) and negatively correlated with HbA1c, blood calcium (Ca), blood phosphorus (P), and 25-hydroxyvitamin D3 (25-OH-D3). Multiple linear regression analysis demonstrated that PTH was significantly associated with HbA1c (*β* = −1.475, *P*=0.003) and HOMA-*β* (*β* = 0.090, *P*=0.001) after adjusting for age, sex, BMI, season, 25-OH-D3, Ca, and P.

**Conclusion:**

PTH was negatively correlated with HbA1c in the newly diagnosed T2D patients. Our results suggested that the PTH level within the reference range is related to islet *β*-cell function and hyperglycemia.

## 1. Introduction

Parathyroid hormone (PTH) is a hormone composed of 84 amino acids secreted by parathyroid cells. It plays a vital role in mineral and bone metabolism by promoting bone resorption, inhibiting urinary calcium (Ca) loss and accelerating vitamin D activation [1]. Nowadays, accumulating evidences support that PTH is related to insulin sensitivity and gestational hyperglycemia [2, 3]. Patients with either primary or secondary hyperparathyroidism have increased fasting blood glucose and exacerbated insulin resistance [4, 5]. Several research studies have shown that parathyroidectomy could improve glycemic control in diabetic patients with hyperparathyroidism [6–8]. Therefore, the abovementioned studies suggested that PTH might regulate glucose metabolism in the patients with hyperparathyroidism and pathological elevation of PTH adversely affected glucose metabolism.

However, the specific relationship between PTH and glucose metabolism is still controversial in individuals without hyperparathyroidism. Previous studies claimed that PTH was not significantly associated with glucose metabolism parameters in the population with normal glucose tolerance, believing that blood Ca rather than PTH was related to glucose metabolism [9–11]. In T2D patients, some evidence revealed that PTH had a beneficial effect on glucose metabolism and was negatively correlated with glycated hemoglobin (HbA1c) [12, 13]. Meanwhile, another study demonstrated that the T2D patients with the higher PTH levels were more likely to have higher HbA1c [14] levels. The relationship between PTH and glucose metabolism in the patients with T2D has received increasing attention because of the conflicting findings. Thus, we aimed to determine if there is an association between PTH and glucose metabolism in the newly diagnosed T2D patients with no primary or secondary hyperparathyroidism.

## 2. Materials and Methods

### 2.1. Study Population

This study consecutively recruited 387 patients with newly diagnosed T2D from January 2017 to April 2018 at the Department of Endocrinology in Beijing Chao-yang Hospital Affiliated to Capital Medical University. All patients with T2D were inpatients and were diagnosed according to the 2013 American Diabetes Association diagnostic criteria. They had not received antidiabetic drugs and negative for diabetes autoantibodies. No participants had hyperparathyroidism or hypothyroidism. Meanwhile, one hundred and forty-five healthy controls were enrolled from Department of Endocrinology at Beijing Chao-yang Hospital Affiliated to Capital Medical University. Exclusion criteria for the controls and T2D group included the following: (1) PTH was higher than the upper limit of normal (88 pg/ml), (2) severe liver and kidney dysfunction, (3) acute diabetes complications and infection, (4) history of malignant tumors, and (5) taking medications considered to affect PTH (e.g., vitamin D, denosumab, and biphosphonate). We further categorized 387 T2D patients into three subgroups according to their HbA1c tertiles as follows: lower HbA1c group (6.00% < HbA1c ≤ 10.10%), medium HbA1c group (10.10% < HbA1c ≤ 11.10%), and higher HbA1c group (11.10% < HbA1c ≤ 14.90%).

This study was conducted according to the ethical standards of the Declaration of Helsinki. All enrolled participants signed informed consent. The protocol of this study was approved by the Ethics Committee of Beijing Chao-yang Hospital Affiliated to Capital Medical University.

### 2.2. Measurement Indicators

We collected basic information about all included individuals, such as age, gender, health status, and medication history. Height and body weight were measured to the nearest 0.1 cm and 0.1 kg by the same trained group, respectively. Also, the body mass index (BMI) was calculated as weight in kilograms (kg) divided by height in meters squared (m^2^).

Venous blood samples were collected at 8.00-9.00 a.m. after overnight fasting for at least 12 hours and tested in the Central Laboratory of Beijing Chao-yang Hospital. Parameters including total cholesterol (TC), low-density lipoprotein cholesterol (LDL-C), high-density lipoprotein cholesterol (HDL-C), triglycerides (TG), free fatty acids (FFA), fasting blood glucose (FBG), liver and kidney function (alanine aminotransferase, aspartate aminotransferase, and creatinine), Ca, and blood phosphorus (P) were measured by Dade-Behring Dimension RXL Auto analyzer (Dade Behring Diagnostics, Marburg, Germany). Fasting plasma insulin (FINS) was measured by Beckman Access 2 (Fullerton, CA). HbA1c was detected by the HLC-723G7 analyzer (Tosoh Corporation, Japan). PTH and 25-hydroxyvitamin D3 (25-OH-D3) were detected by Enzyme-linked immunosorbent assay (ELISA). Homeostasis model assessment of insulin resistance (HOMA-IR) and homeostasis model assessment of *β*-cell function (HOMA-*β*) were calculated according to the following formulas: HOMA-IR = FINS (*μ*IU/mL) × FBG (mmol/L)/22.5 and HOMA-*β* = 20 × FINS (*μ*IU/mL)/[FBG (mmol/L) − 3.5)].

### 2.3. Statistical Analysis

All statistical analyses were performed using SPSS 26.0 (IBM Corporation, New York). The Kolmogorov–Smirnov test was used to evaluate the distribution of continuous data. For normally distributed data, continuous data were reported as the mean ± standard deviation. Also, non-normally distributed data were expressed as the median (P25, P75). The differences between the two groups were compared using an independent sample *t*-test or a Mann–Whitney *U* test. While the ANOVA test or the Kruskal–Wallis H test was used to compare the differences among the three groups, and intergroup differences were analyzed with the Bonferroni post hoc test. The Chi-square test was used for categorical variables. The Spearman rank correlation test was used for the analysis of bivariate correlation. Multivariate linear regression analysis was performed to assess the relationship between PTH and relevant variables. Statistical significance was considered with two-tailed analyses as *P* < 0.05.

## 3. Results

### 3.1. Baseline Characteristics of the T2D and Control Groups

**Figure 1:**
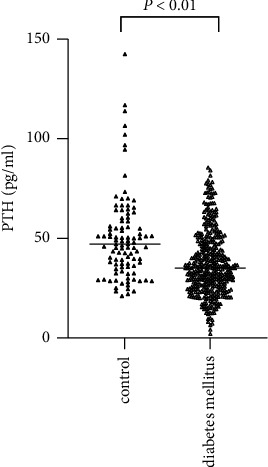
PTH levels of the newly diagnosed T2D and the control groups. PTH: parathyroid hormone; T2D: type 2 diabetes.

The baseline characteristics of the T2D and control groups are presented in Table 1. Age was comparable in the two groups (*P* > 0.05). There were no significant differences in FINS, TC, LDL-C, and FFA between the two groups. The T2D patients had significantly higher BMI, TG, and P levels and lower HDL-C and Ca levels compared with the controls (*P* < 0.001). However, the PTH levels were lower in the T2D group than those in the control group ((*P* < 0.001); Figure 1).

### 3.2. Baseline Characteristics of the T2D Patients with Different HbA1c Levels

**Figure 2:**
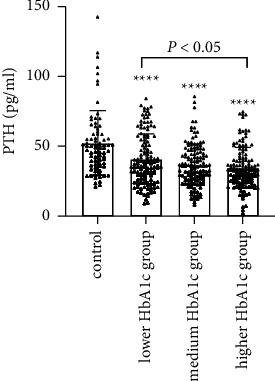
PTH levels of the newly diagnosed T2D patients with different HbA1c levels. PTH: parathyroid hormone; T2D: type 2 diabetes. Compared with the control group, ^*∗∗∗∗*^significantly different at *P* < 0.001.

We further divided 387 newly diagnosed T2D patients into the three subgroups according to the HbA1c tertiles in Table 2. There were no significant differences in age, sex, BMI, FINS, LDL-C, TG, FFA, Ca, P, 25-OH-D3, and the season of blood sample collection among the three groups (*P* > 0.05). The T2D patients with a higher HbA1c tertile had higher HOMA-IR and lower HOMA-*β* levels than the patients with a lower HbA1c tertile (HOMA-IR: *P* < 0.01, HOMA-*β*: *P* < 0.001). Interestingly, PTH levels in the patients with a higher HbA1c tertile were significantly lower than the patients with a lower HbA1c tertile (*P* < 0.05; Figure 2).

### 3.3. The Correlation between PTH and Clinical Parameters in the Patients with Newly Diagnosed T2D

**Figure 3:**
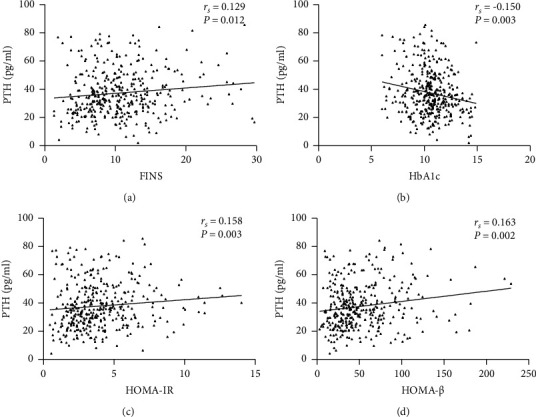
The correlation between PTH and other parameters in the newly diagnosed T2D patients. (a) Correlation between PTH and FINS. (b) Correlation between PTH and HbA1c. (c) Correlation between PTH and HOMA-IR. (d) Correlation between PTH and HOMA-*β*. PTH: parathyroid hormone; T2D: type 2 diabetes; FINS: fasting plasma insulin; HbA1c: glycated hemoglobin; HOMA-IR: homeostasis model assessment of insulin resistance; HOMA-*β*: homeostasis model assessment of *β*-cell function.

Spearman correlation analysis was used to explore the relationship between PTH and other parameters in the patients with newly diagnosed T2D (Table 3). PTH was not related to age, FBG, TC, LDL-C, HDL-C, TG, and FFA. PTH was positively associated with BMI (*r*_*s*_ = 0.166, *P* = 0.001), FINS (*r*_*s*_ = 0.129, *P* < 0.05; Figure 3(a)), HOMA-IR (*r*_*s*_ = 0.158, *P* < 0.01; Figure 3(c)), and HOMA-*β* (*r*_*s*_ = 0.163, *P* < 0.01; Figure 3(d)), and it was negatively related to HbA1c (*r*_*s*_ = −0.150, *P* < 0.01; Figure 3(b)), Ca (*r*_*s*_ = −0.179, *P* < 0.001), P (*r*_*s*_ = −0.180, *P* < 0.001), and 25-OH-D3 (*r*_*s*_ = −0.246, *P* < 0.001). Finally, multiple regression analysis was performed to investigate whether PTH levels were related to glucose metabolism parameters in the T2D patients, including HbA1c, HOMA-IR, and HOMA-*β* in Table 4. After adjusting for age, sex, BMI, season, 25-OH-D3, Ca, and P, PTH was negatively correlated with HbA1c (*β* = −1.475, *P* < 0.05).

## 4. Discussion

The current study showed that the PTH levels were significantly lower in the patients with newly diagnosed T2D compared with the controls. Besides, the T2D patients with a higher HbA1c tertile had lower PTH levels. Spearman correlation analysis showed that the PTH was positively correlated with FINS, HOMA-IR, and HOMA-*β* while negatively correlated with HbA1c. Multiple regression analysis showed that PTH was independently associated with HbA1c and HOMA-*β*.

In the present study, the patients with T2D had higher P, lower Ca, and lower 25-OH-D than the controls. In these conditions, PTH levels might increase within the reference range, whereas their PTH levels were still lower than the controls. In line with that, previous studies claimed that PTH levels were lower in the patients with newly diagnosed T2D compared with the controls [11, 15, 16]. These suggest that PTH might be correlated with glucose metabolism. In order to explore the relationship between different blood glucose states and PTH levels, we further evaluated PTH levels of the T2D patients in different HbA1c levels with the comparable Ca, P, and 25-OH-D3 levels. It was interesting that PTH levels in the higher HbA1c group were significantly lower than in the lower HbA1c group. Further correction analysis and multiple regression analysis found consistent results with previous studies and showed that PTH was positively correlated with HOMA-*β* while negatively correlated with HbA1c in the patients with newly diagnosed T2D [12, 17]. Thus, we speculated that the PTH level within the reference range is related to islet *β*-cell function and hyperglycemia. Human *β*-cell expresses parathyroid hormone-1 receptor [18]. A recent study preformed that PTH-related peptide could significantly enhance *β*-cell regeneration after partial pancreatectomy in a mouse model [19]. Further support for PTH-receptor interacting in the islet is provided by the previous observation that PTH facilitates glucose-induced insulin release by increasing the cytosolic Ca [20] and stimulating the production of cyclic adenosine mono phosphate (cAMP) [3]. Sahai et al. found that PTH stimulates protein kinase C (PKC) activity of islet in a manner similar to glucose for insulin secretion [21]. Instead, some researchers supposed that the hyperglycemia condition leading to lower PTH level. An experiment in vitro on bovine parathyroid cells found that high glucose concentration and lacking insulin could suppress PTH secretion [22]. Also, PTH secretion could be recovered in a low glucose concentration [22]. In line with this concept, hyperglycemia-induced inflammation could inhibit the secretion of PTH [16]. Existing evidence revealed that magnesium level could decrease under hyperglycemia, with a mechanism of reducing the net tubular reabsorption of it and consequently less PTH secretion [23, 24]. Epidemiological studies have shown that a 2- to 4-fold increase of the prevalence of diabetes in primary hyperparathyroidism compared with general population [25, 26]. However, these studies did not pay attention to the T2D patients with normal parathyroid function, which was different from our results. Thus, more comprehensive experiments could be carried out in the future to further explore the mechanism.

In summary, our study demonstrated that the T2D group with higher HbA1c levels has lower normal range PTH levels. Previous studies have shown that the higher normal PTH level might have a positive effect on islet *β*-cell function, and in turn, high glucose environment could inhibit the secretion of PTH, which guides that clinical workers should pay more attention to PTH levels in the patients with T2D. On the one hand, a higher PTH level within the reference range might improve the islet *β*-cell function. On the other hand, poor glucose condition could increase the risk of hypoparathyroidism.

Our research has some limitations. First, the direct causal relationship between PTH and HbA1c cannot be inferred from the observational association in this study. Second, due to limitations of the data collection, our study did not control for exercise and diet and did not include data on Ca intake and urinary Ca excretion. Third, the newly diagnosed T2D patients were inpatients leading to poor glycemic compensation. Finally, our research was a small-scale study, and large-scale cohort studies are needed to validate our results in the future.

## 5. Conclusions

PTH was negatively correlated with HbA1c in the newly diagnosed T2D patients. Our results suggested that the PTH level within the reference range is related to islet *β*-cell function and hyperglycemia.

## Figures and Tables

**Table 1:** Baseline characteristics of the newly diagnosed T2D patients and the controls.

	Control (*n* = 145)	T2D (*n* = 387)	*P*
Age (years)	43.87 ± 12.39	45.51 ± 11.59	0.155
Sex, male (*n*) %	13 (14.8%)	281 (72.6%)	<0.001
Symptomatic (*n*) %	0 (0%)	216 (55.8%)	<0.001
BMI (kg/m^2^)	24.75 ± 3.89	26.86 ± 3.33	<0.001
TC (mmol/L)	4.98 ± 0.97	4.93 ± 0.93	0.572
LDL-C (mmol/L)	3.23 ± 1.19	3.12 ± 0.94	0.260
HDL-C (mmol/L)	1.35 ± 0.34	0.97 ± 0.21	<0.001
TG (mmol/L)	1.19 (0.88, 1.80)	1.76 (1.26, 2.60)	<0.001
FFA (mmol/L)	0.56 ± 0.23	0.53 ± 0.18	0.056
Crea (*μ*mol/L)	53.70 (49.10, 60.30)	59.80 (49.52, 69.45)	0.001
FBG (mmol/L)	5.00 (4.74, 5.32)	7.94 (6.63, 9.81)	<0.001
FINS (*μ*IU/mL)	9.30 (6.35, 14.40)	10.10 (7.00, 13.20)	0.560
HbA1c (%)	5.50 (5.28, 5.40)	10.60 (9.70, 11.60)	<0.001
HOMA-IR	1.65 (0.99, 3.15)	3.49 (2.36, 5.09)	<0.001
HOMA-*β*	114.94 (66.30, 161.36)	41.88 (28.51, 69.34)	<0.001
Ca (mmol/L)	2.37 ± 0.09	2.31 ± 0.10	<0.001
P (mmol/L)	1.23 ± 0.15	1.32 ± 0.20	<0.001
25-OH-D3 (ng/mL)	17.40 (11.81, 23.71)	14.64 (10.93, 19.12)	0.007
PTH (pg/mL)	47.15 (35.83, 58.65)	35.10 (25.90, 47.20)	<0.001

Data are shown as the means ± S.D. unless indicated otherwise. TG, FBG, FINS, HbA1c, HOMA-IR, HOMA-*β*, 25-OH-D3, and PTH are shown as the median (P25 and P75). Male and season are shown as *n* (%). Symptomatic: polydipsia, polyuria, and loss of weight; BMI, body mass index; TC, total cholesterol; LDL-C, low-density lipoprotein cholesterol; HDL-C, high-density lipoprotein cholesterol; TG, triglycerides; FFA, free fatty acids; Crea, creatinine; FBG, fasting blood glucose; FINS, fasting plasma insulin; HbA1c, glycated hemoglobin; HOMA-IR, homeostasis model assessment of insulin resistance; HOMA-*β*, homeostasis model assessment of *β*-cell function; Ca, blood calcium; P, blood phosphorus; 25-OH-D3, 25-hydroxyvitamin D3; PTH, parathyroid hormone.

**Table 2:** Baseline characteristics of the newly diagnosed T2D patients with various levels of HbA1c.

	Tertile of HbA1c(*n* = 387)			*P*
	Lower HbA1c group (*n* = 129)	Medium HbA1c group (*n* = 129)	Higher HbA1c group (*n* = 129)	
Age (years)	46.7 ± 11.1	45.8 ± 11.7	43.9 ± 11.7	0.144
Sex, male, (*n*) %	98 (75.9%)	93 (72.0%)	90 (69.7%)	0.529
Symptomatic (*n*) %	56 (43.4%)	70 (54.3%)^a^	90 (69.8%)^ab^	<0.001
Season, summer, (*n*) %	34 (26.3%)	33 (25.5%)	35 (27.1%)	0.961
BMI (kg/m^2^)	27.12 ± 3.20	26.84 ± 3.33	26.60 ± 3.45	0.451
TC (mmol/L)	4.82 ± 0.74	4.84 ± 0.94	5.11 ± 1.04^a^	0.024
LDL-C (mmol/L)	3.05 ± 0.77	3.05 ± 0.88	3.23 ± 1.11	0.211
HDL-C (mmol/L)	0.99 (0.80, 1.10)	0.94 (0.80, 1.10)	0.90 (0.79, 1.00)	0.041
TG (mmol/L)	1.76 (1.34, 2.52)	1.78 (1.18, 2.63)	1.75 (1.31, 2.68)	0.733
FFA (mmol/L)	0.52 (0.42, 0.66)	0.50 (0.38, 0.61)	0.49 (0.38, 0.63)	0.063
Crea (*μ*mol/L)	64.10 (52.90, 74.10)	57.60 (47.20, 66.10)^a^	57.80 (49.40, 66.80)^a^	<0.001
FBG (mmol/L)	7.18 (6.05, 8.29)	8.00 (6.59, 9.57)^a^	9.35 (7.41, 11.45)^ab^	<0.001
FINS (*μ*IU/mL)	9.30 (6.95, 12.92)	10.30 (7.00, 13.90)	10.10 (7.20, 12.90)	0.705
HbA1c (%)	9.00 (8.15, 9.70)	10.60 (10.40, 10.80)^a^	12.30 (11.55, 12.95)^ab^	<0.001
HOMA-IR	3.03 (2.03, 4.39)	3.50 (2.46, 5.20)	3.98 (2.86, 5.41)^a^	0.001
HOMA-*β*	54.21 (34.92, 76.29)	40.31 (27.54, 68.83)	35.08 (21.85, 56.90)^a^	<0.001
Ca (mmol/L)	2.30 ± 0.09	2.31 ± 0.09	2.30 ± 0.11	0.728
P (mmol/L)	1.29 ± 0.20	1.34 ± 0.20	1.31 ± 0.20	0.126
25-OH-D3 (ng/mL)	15.63 (11.05, 18.77)	14.20 (10.90, 19.60)	13.93 (10.73, 20.89)	0.773
PTH (pg/mL)	37.50 (26.10, 54.55)	36.00 (26.65, 46.50)	32.90 (24.85, 41.40)^a^	0.031

Data are shown as the means ± S.D. unless indicated otherwise. HDL-C, TG, FFA, FBG, FINS, HbA1c, HOMA-IR, HOMA-*β*, 25-OH-D3, and PTH are shown as the median (P25 and P75). Male and season are shown as *n* (%). HbA1c, glycated hemoglobin; symptomatic: polydipsia, polyuria and loss of weight; BMI, body mass index; TC, total cholesterol; LDL-C, low-density lipoprotein cholesterol; HDL-C, high-density lipoprotein cholesterol; TG, triglycerides; FFA, free fatty acids; Crea, creatinine; FBG, fasting blood glucose; FINS, fasting plasma insulin; HOMA-IR, homeostasis model assessment of insulin resistance; HOMA-*β*, homeostasis model assessment of *β*-cell function; Ca, blood calcium; P, blood phosphorus; 25-OH-D3, 25-hydroxyvitamin D3; PTH, parathyroid hormone. Compared with the lower HbA1c group, ^a^significantly different at *P* < 0.05. Compared with the medium HbA1c group, ^b^significantly different at *P* < 0.05.

**Table 3:** Spearman correlation analysis between PTH and other parameters in the newly diagnosed T2D patients.

Parameters	PTH	
	*r*	*P*
Age (years)	−0.024	0.632
BMI (kg/m^2^)	0.166	0.001
Ca (mmol/L)	−0.179	<0.001
P (mmol/L)	−0.180	<0.001
25-OH-D3 (ng/mL)	−0.246	<0.001
TC (mmol/L)	−0.067	0.190
LDL-C (mmol/L)	−0.045	0.376
HDL-C (mmol/L)	0.019	0.706
TG (mmol/L)	0.002	0.965
FFA (mmol/L)	0.095	0.064
Crea (*μ*mol/L)	−0.003	0.959
HbA1c (%)	−0.150	0.003
FBG (mmol/L)	−0.040	0.445
FINS (*μ*IU/mL)	0.129	0.012
HOMA-*β*	0.163	0.002
HOMA-IR	0.158	0.003

PTH, parathyroid hormone; BMI, body mass index; Ca, blood calcium; P, blood phosphorus; 25-OH-D3, 25-hydroxyvitamin D3; TC, total cholesterol; LDL-C, low-density lipoprotein cholesterol; HDL-C, high-density lipoprotein cholesterol; TG, triglycerides; FFA, free fatty acids; Crea, creatinine; HbA1c, glycated hemoglobin; FBG, fasting blood glucose; FINS, fasting plasma insulin; HOMA-*β*, homeostasis model assessment of *β*-cell function; HOMA-IR, homeostasis model assessment of insulin resistance.

**Table 4:** Multiple regression analysis between PTH and other parameters in newly diagnosed type 2 diabetic patients.

	Model 1			Model 2		
	*β*	95% CI	*P*	*β*	95% CI	*P*
HbA1c	−1.708	(−2.631, −0.784)	<0.001	−1.475	(−2.460, −0.490)	0.003
HOMA-IR	0.741	(0.007, 1.475)	0.048	0.093	(−0.769, 0.955)	0.831
HOMA-*β*	0.072	(0.027, 0.118)	0.002	0.090	(0.039, 0.141)	0.001

PTH, parathyroid hormone; HbA1c, glycated hemoglobin; HOMA-IR, homeostasis model assessment of insulin resistance; HOMA-*β*, homeostasis model assessment of *β*-cell function. Model 1: values of *β* and 95% confidence interval (CI) were calculated using the multivariate linear regression models without adjustment for variables. Model 2: values of *β* and 95% CI were calculated using the multivariate linear regression models and adjusted for age, sex, season, BMI, 25-OH-D3, Ca, and P.

## Data Availability

The data used to support the findings of this study are included within the article.
